# NaFe(TeO_3_)_2_

**DOI:** 10.1107/S1600536807065403

**Published:** 2007-12-06

**Authors:** Matthias Weil, Berthold Stöger

**Affiliations:** a Institute for Chemical Technologies and Analytics, Division of Structural Chemistry, Vienna University of Technology, Getreidemarkt 9/164-SC, A-1060 Vienna, Austria

## Abstract

The hydro­thermally prepared title compound, sodium iron(III) bis­[trioxotellurate(IV)], is isotypic with its Ga^III^ analogue and consists of corrugated layers with an overall composition of [FeTe_2_O_6_]^−^ together with Na^+^ cations. The layers extend parallel to (001) and are made up of [Fe_2_O_10_] edge-shared octa­hedral dimers and TeO_3_ trigonal pyramids sharing vertices. The Na^+^ cations are located in the cavities of this arrangement and link adjacent [FeTe_2_O_6_]^−^ layers *via* distorted [NaO_8_] polyhedra.

## Related literature

For the isotypic structure NaGa(TeO_3_)_2_, see: Miletich & Pertlik (1998 ▶). For related structures, see: Weil (2005 ▶, 2007 ▶); Weil & Stöger (2007 ▶). For a review on the crystal chemistry of tellurate(IV) oxocompounds, see: Dolgikh (1991 ▶).

## Experimental

### 

#### Crystal data

NaFe(TeO_3_)_2_
*M* *_r_* = 430.04
Orthorhombic, 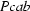

*a* = 7.8530 (15) Å
*b* = 10.448 (2) Å
*c* = 13.438 (3) Å
*V* = 1102.5 (4) Å^3^
*Z* = 8
Mo *K*α radiation
μ = 13.15 mm^−1^
*T* = 293 (2) K
0.08 × 0.02 × 0.01 mm

#### Data collection

Bruker SMART APEX CCD diffractometer
Absorption correction: multi-scan (*SADABS*; Bruker, 2002 ▶) *T*_min_ = 0.405, *T*_max_ = 0.858
11127 measured reflections
1598 independent reflections
1329 reflections with *I* > 2σ(*I*)
*R*_int_ = 0.053

#### Refinement

*R*[*F* ^2^ > 2σ(*F* ^2^)] = 0.027
*wR*(*F* ^2^) = 0.061
*S* = 1.03
1598 reflections
91 parameters
Δρ_max_ = 1.77 e Å^−3^
Δρ_min_ = −0.96 e Å^−3^

### 

Data collection: *SMART* (Bruker, 2002 ▶); cell refinement: *SAINT* (Bruker, 2002 ▶); data reduction: *SAINT*; method used to solve structure: coordinates taken from an isotypic structure; program(s) used to refine structure: *SHELXL97* (Sheldrick, 1997 ▶); molecular graphics: *ATOMS* (Dowty, 2006 ▶); software used to prepare material for publication: *SHELXL97*.

## Supplementary Material

Crystal structure: contains datablocks I, global. DOI: 10.1107/S1600536807065403/hb2673sup1.cif

Structure factors: contains datablocks I. DOI: 10.1107/S1600536807065403/hb2673Isup2.hkl

Additional supplementary materials:  crystallographic information; 3D view; checkCIF report

## Figures and Tables

**Table 1:** Selected bond lengths (Å)

Na—O5^i^	2.434 (4)
Na—O6^ii^	2.436 (4)
Na—O3^ii^	2.491 (4)
Na—O2^iii^	2.581 (5)
Na—O2^ii^	2.755 (5)
Na—O1^i^	2.758 (4)
Na—O4^i^	2.788 (4)
Na—O3^iii^	2.958 (4)
Fe—O3^iv^	1.942 (4)
Fe—O1^v^	1.955 (4)
Fe—O5^vi^	2.036 (3)
Fe—O6	2.037 (4)
Fe—O4^vii^	2.055 (4)
Fe—O4^vi^	2.078 (4)
Te1—O1	1.893 (4)
Te1—O3^ii^	1.901 (4)
Te1—O4	1.901 (4)
Te2—O2^viii^	1.849 (4)
Te2—O6^ix^	1.892 (4)
Te2—O5^x^	1.899 (4)

Symmetry codes: (i) 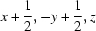; (ii) 
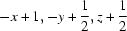
; (iii) 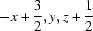; (iv) 
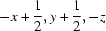
; (v) 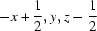; (vi) 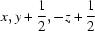; (vii) 
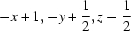
; (viii) 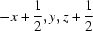; (ix) 
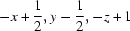
; (x) 
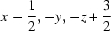
.

**Fig. 1.**
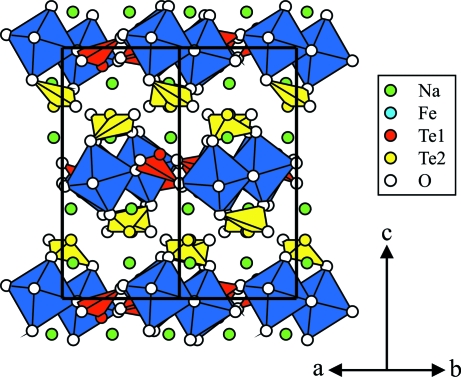
The crystal structure of NaGa(TeO3)2 in projection along [110].

**Fig. 2.**
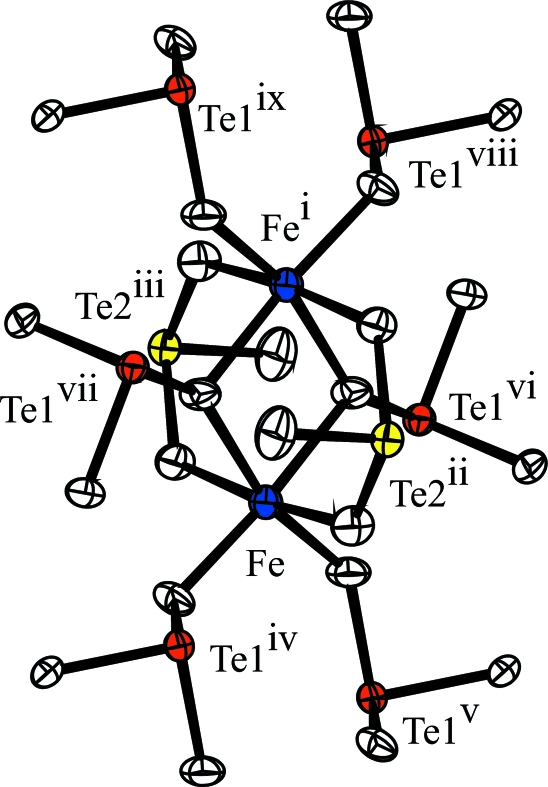
The [Fe2O10] dimer with the corner-sharing TeO3 trigonal-pyramids attached. Atoms are drawn as displacement ellipsoids at the 90% probability level. [Symmetry operators: (i) -x + 1, -y + 1, z; (ii) -x + 1/2, y + 1/2, -z + 1; (iii) x + 1/2, -y + 1/2, z - 1; (iv) x - 1/2, -y + 1, -z + 1/2; (v) -x + 1/2, -y + 1/2, z - 1; (vi) -x + 1, -y + 1/2, z - 1/2; (vii) x, y + 1/2, -z + 1/2; (viii) -x + 1.5, y, z - 1/2; (ix) x + 1/2, -y + 1, -z + 1/2.]

## supplementary crystallographic information

### Comment

The present communication is part of our ongoing studies of the phase formation and structures of Te(IV)-containing oxocompounds formed under hydrothermal conditions (*e.g.* Weil, 2005, 2007; Weil & Stöger, 2007).

The crystal structure of the title compound, (I), is built up of layers with an overall composition [FeTe_2_O_6_]^-^ extending parallel to (001). Adjacent layers are linked by Na^+^ cations that are located in the voids of this arrangement (Fig. 1).

The anionic layers consists of octahedral [FeO_6_] and trigonal-pyramidal TeO_3_ units as simple building blocks (Table 1). Two edge-sharing [FeO_6_] octahedra [mean Fe—O = 2.017 Å] form a centrosymmetric [Fe_2_O_10_] dimer which is connected to eight TeO_3_ units *via* oxygen-atom corners. The equatorial oxygen atoms of the dimer are linked to six Te1O_3_ groups whereas the axial oxygen atoms of the dimer are part of two Te2O_3_ groups capping both Fe atoms at the top and at the bottom (Fig. 2). Each of the free corners of the Te1O_3_ groups are further linked to adjacent [Fe_2_O_10_] dimers thus establishing the layered arrangement. The lone-pair electrons of the tellurium(IV) atoms point towards the free space and are aligned approximately parallel to [001]. The Na^+^ cations are surrounded by eight oxygen atoms, leading to distorted polyhedra with a mean Na—O of 2.650 Å. The Te—O bond lengths and mean O—Te—O angle of 96.3° are typical values for trigonal-pyramidal TeO_3_ units (Dolgikh, 1991). The next nearest O sites relative to the Te centres are outside of the first coordination spheres with distances of Te1—O2 = 2.549 (4) Å, Te1—O1[*x* + 1/2, -*y* + 1/2, *z*] = 2.570 (4) Å and Te2—O5 = 2.703 (4) Å.

The crystal structure of NaFe(TeO_3_)_2_ is isotypic with the Ga^III^ analogue, NaGa(TeO_3_)_2_ (Miletich & Pertlik, 1998), and exhibits similar interatomic distances and angles.

### Experimental

All chemicals used were of analytical grade (Merck, p.A.) and employed without further purification: 20 mg (0.5 mmol) NaOH, 53 mg (0.33 mmol) Fe_2_O_3_ and 160 mg (1 mmol) TeO_2_ were placed in a 5-ml Teflon-lined steel autoclave that was filled with 2 ml demineralized water. The autoclave was heated at 493 K for 6 d and then cooled to room temperature within 3 h. The reaction product consisted mainly of a mixture of unreacted Fe_2_O_3_ and TeO_2_. Only few colourless crystals of (I) with unspecific habit were obtained.

### Refinement

For better comparison with the isotypic NaGa(TeO_3_)_2_ structure, the refinement was carried out in the non-standard setting *Pcab* of space group No. 61 (standard setting *Pbca*). The atomic coordinates of the Ga analogue were taken as starting parameters. The highest remaining peak in the final difference Fourier map is 0.71 Å from Te2 and the deepest hole is 0.79 Å from O4.

### Crystal data

**Table:**

NaFe(TeO_3_)_2_	*F*_000_ = 1512
*M**_r_* = 430.04	*D*_x_ = 5.182 Mg m^−^^3^
Orthorhombic, *P**c**a**b*	Mo *K*α radiation λ = 0.71073 Å
Hall symbol: -P 2bc 2ac	Cell parameters from 2569 reflections
*a* = 7.8530 (15) Å	θ = 3.0–30.0º
*b* = 10.448 (2) Å	µ = 13.15 mm^−^^1^
*c* = 13.438 (3) Å	*T* = 293 (2) K
*V* = 1102.5 (4) Å^3^	Prism, colourless
*Z* = 8	0.08 × 0.02 × 0.01 mm

### Data collection

**Table:**

Bruker SMART APEX CCD diffractometer	1598 independent reflections
Radiation source: fine-focus sealed tube	1329 reflections with *I* > 2σ(*I*)
Monochromator: graphite	*R*_int_ = 0.053
*T* = 293(2) K	θ_max_ = 30.0º
ω scans	θ_min_ = 3.0º
Absorption correction: multi-scan(SADABS; Bruker, 2002)	*h* = −11→9
*T*_min_ = 0.405, *T*_max_ = 0.858	*k* = −14→14
11127 measured reflections	*l* = −18→18

### Refinement

**Table:**

Refinement on *F*^2^	Primary atom site location: isomorphous structure methods
Least-squares matrix: full	*w* = 1/[σ^2^(*F*_o_^2^) + (0.0323*P*)^2^] where *P* = (*F*_o_^2^ + 2*F*_c_^2^)/3
*R*[*F*^2^ > 2σ(*F*^2^)] = 0.027	(Δ/σ)_max_ < 0.001
*wR*(*F*^2^) = 0.061	Δρ_max_ = 1.77 e Å^−^^3^
*S* = 1.03	Δρ_min_ = −0.96 e Å^−^^3^
1598 reflections	Extinction correction: none
91 parameters	

### Special details

**Table:**

Geometry. All e.s.d.'s (except the e.s.d. in the dihedral angle between two l.s. planes) are estimated using the full covariance matrix. The cell e.s.d.'s are taken into account individually in the estimation of e.s.d.'s in distances, angles and torsion angles; correlations between e.s.d.'s in cell parameters are only used when they are defined by crystal symmetry. An approximate (isotropic) treatment of cell e.s.d.'s is used for estimating e.s.d.'s involving l.s. planes.
Refinement. Refinement of *F*^2^ against ALL reflections. The weighted *R*-factor *wR* and goodness of fit *S* are based on *F*^2^, conventional *R*-factors *R* are based on *F*, with *F* set to zero for negative *F*^2^. The threshold expression of *F*^2^ > σ(*F*^2^) is used only for calculating *R*-factors(gt) *etc*. and is not relevant to the choice of reflections for refinement. *R*-factors based on *F*^2^ are statistically about twice as large as those based on *F*, and *R*- factors based on ALL data will be even larger.

### Fractional atomic coordinates and isotropic or equivalent isotropic displacement parameters (Å^2^)

**Table:**

	*x*	*y*	*z*	*U*_iso_*/*U*_eq_	
Te1	0.54652 (4)	0.28714 (3)	0.42454 (2)	0.00780 (9)	
Te2	0.08184 (4)	0.00810 (3)	0.76667 (2)	0.00932 (9)	
Fe	0.30472 (10)	0.49980 (6)	−0.02064 (5)	0.00866 (15)	
Na	0.8320 (3)	0.2417 (2)	0.64216 (19)	0.0254 (6)	
O1	0.3210 (4)	0.3435 (3)	0.4480 (3)	0.0122 (7)	
O2	0.4293 (5)	0.1849 (4)	0.2665 (3)	0.0169 (8)	
O3	0.3696 (4)	0.1292 (3)	0.0402 (3)	0.0106 (7)	
O4	0.4983 (4)	0.1292 (3)	0.4888 (3)	0.0104 (7)	
O5	0.3769 (5)	0.0296 (3)	0.6643 (3)	0.0107 (7)	
O6	0.2625 (5)	0.4796 (3)	0.1281 (3)	0.0119 (7)	

### Atomic displacement parameters (Å^2^)

**Table:**

	*U*^11^	*U*^22^	*U*^33^	*U*^12^	*U*^13^	*U*^23^
Te1	0.00751 (16)	0.00640 (14)	0.00950 (15)	−0.00033 (11)	0.00003 (11)	0.00063 (11)
Te2	0.00962 (16)	0.00905 (15)	0.00928 (16)	0.00021 (11)	0.00013 (11)	−0.00144 (11)
Fe	0.0085 (3)	0.0069 (3)	0.0106 (4)	0.0000 (3)	−0.0002 (3)	−0.0005 (2)
Na	0.0326 (15)	0.0142 (11)	0.0294 (13)	−0.0056 (10)	−0.0082 (11)	0.0034 (10)
O1	0.0065 (17)	0.0113 (16)	0.0188 (19)	0.0005 (14)	0.0010 (14)	−0.0019 (14)
O2	0.022 (2)	0.0110 (17)	0.0175 (19)	−0.0020 (15)	−0.0020 (16)	0.0000 (14)
O3	0.0087 (17)	0.0094 (16)	0.0138 (17)	−0.0030 (14)	−0.0017 (14)	0.0023 (13)
O4	0.0074 (17)	0.0085 (16)	0.0152 (17)	0.0012 (13)	−0.0020 (13)	0.0028 (13)
O5	0.0096 (17)	0.0156 (17)	0.0071 (17)	0.0007 (14)	−0.0014 (13)	−0.0017 (14)
O6	0.0119 (18)	0.0154 (17)	0.0084 (17)	−0.0003 (14)	0.0003 (14)	−0.0006 (13)

### Geometric parameters (Å, °)

**Table:**

Na—O5^i^	2.434 (4)	Fe—O5^vi^	2.036 (3)
Na—O6^ii^	2.436 (4)	Fe—O6	2.037 (4)
Na—O3^ii^	2.491 (4)	Fe—O4^vii^	2.055 (4)
Na—O2^iii^	2.581 (5)	Fe—O4^vi^	2.078 (4)
Na—O2^ii^	2.755 (5)	Te1—O1	1.893 (4)
Na—O1^i^	2.758 (4)	Te1—O3^ii^	1.901 (4)
Na—O4^i^	2.788 (4)	Te1—O4	1.901 (4)
Na—O3^iii^	2.958 (4)	Te2—O2^viii^	1.849 (4)
Fe—O3^iv^	1.942 (4)	Te2—O6^ix^	1.892 (4)
Fe—O1^v^	1.955 (4)	Te2—O5^x^	1.899 (4)
			
O1—Te1—O3^ii^	92.59 (15)	O6^ii^—Na—O4^i^	123.02 (15)
O1—Te1—O4	90.44 (15)	O3^ii^—Na—O4^i^	68.23 (12)
O3^ii^—Te1—O4	95.54 (16)	O2^iii^—Na—O4^i^	104.45 (14)
O2^viii^—Te2—O6^ix^	100.85 (16)	O2^ii^—Na—O4^i^	131.48 (14)
O2^viii^—Te2—O5^x^	99.64 (16)	O1^i^—Na—O4^i^	58.10 (12)
O6^ix^—Te2—O5^x^	98.64 (16)	O5^i^—Na—O3^iii^	109.39 (14)
O3^iv^—Fe—O1^v^	101.34 (15)	O6^ii^—Na—O3^iii^	80.11 (13)
O3^iv^—Fe—O5^vi^	87.79 (15)	O3^ii^—Na—O3^iii^	117.64 (17)
O1^v^—Fe—O5^vi^	93.64 (15)	O2^iii^—Na—O3^iii^	68.48 (13)
O3^iv^—Fe—O6	95.17 (15)	O2^ii^—Na—O3^iii^	168.98 (14)
O1^v^—Fe—O6	92.48 (15)	O1^i^—Na—O3^iii^	57.21 (11)
O5^vi^—Fe—O6	172.55 (15)	O4^i^—Na—O3^iii^	58.59 (11)
O3^iv^—Fe—O4^vii^	174.47 (15)	Te1—O1—Fe^viii^	140.2 (2)
O1^v^—Fe—O4^vii^	81.12 (15)	Te1—O1—Na^xi^	91.60 (15)
O5^vi^—Fe—O4^vii^	87.11 (14)	Fe^viii^—O1—Na^xi^	94.66 (14)
O6—Fe—O4^vii^	89.65 (14)	Te2^v^—O2—Na^xii^	105.36 (18)
O3^iv^—Fe—O4^vi^	95.20 (15)	Te2^v^—O2—Na^vii^	104.09 (17)
O1^v^—Fe—O4^vi^	163.16 (15)	Na^xii^—O2—Na^vii^	94.82 (15)
O5^vi^—Fe—O4^vi^	83.80 (14)	Te1^vii^—O3—Fe^xiii^	116.98 (18)
O6—Fe—O4^vi^	89.11 (14)	Te1^vii^—O3—Na^vii^	114.90 (17)
O4^vii^—Fe—O4^vi^	82.13 (15)	Fe^xiii^—O3—Na^vii^	90.19 (14)
O5^i^—Na—O6^ii^	170.30 (17)	Te1^vii^—O3—Na^xii^	85.51 (13)
O5^i^—Na—O3^ii^	68.14 (13)	Fe^xiii^—O3—Na^xii^	153.87 (17)
O6^ii^—Na—O3^ii^	106.13 (15)	Na^vii^—O3—Na^xii^	91.93 (13)
O5^i^—Na—O2^iii^	92.37 (15)	Te1—O4—Fe^ii^	113.01 (17)
O6^ii^—Na—O2^iii^	93.05 (14)	Te1—O4—Fe^xiv^	143.49 (19)
O3^ii^—Na—O2^iii^	160.49 (15)	Fe^ii^—O4—Fe^xiv^	97.87 (15)
O5^i^—Na—O2^ii^	76.12 (14)	Te1—O4—Na^xi^	90.52 (14)
O6^ii^—Na—O2^ii^	94.86 (14)	Fe^ii^—O4—Na^xi^	135.71 (17)
O3^ii^—Na—O2^ii^	73.11 (14)	Fe^xiv^—O4—Na^xi^	79.65 (12)
O2^iii^—Na—O2^ii^	102.25 (16)	Te2^xv^—O5—Fe^xiv^	131.84 (19)
O5^i^—Na—O1^i^	115.91 (14)	Te2^xv^—O5—Na^xi^	112.72 (17)
O6^ii^—Na—O1^i^	67.10 (12)	Fe^xiv^—O5—Na^xi^	89.63 (14)
O3^ii^—Na—O1^i^	68.55 (13)	Te2^xvi^—O6—Fe	127.7 (2)
O2^iii^—Na—O1^i^	124.14 (15)	Te2^xvi^—O6—Na^vii^	106.73 (16)
O2^ii^—Na—O1^i^	129.82 (15)	Fe—O6—Na^vii^	102.93 (15)
O5^i^—Na—O4^i^	63.09 (12)		

Symmetry codes: (i) *x*+1/2, −*y*+1/2, *z*; (ii) −*x*+1, −*y*+1/2, *z*+1/2; (iii) −*x*+3/2, *y*, *z*+1/2; (iv) −*x*+1/2, *y*+1/2, −*z*; (v) −*x*+1/2, *y*, *z*−1/2; (vi) *x*, *y*+1/2, −*z*+1/2; (vii) −*x*+1, −*y*+1/2, *z*−1/2; (viii) −*x*+1/2, *y*, *z*+1/2; (ix) −*x*+1/2, *y*−1/2, −*z*+1; (x) *x*−1/2, −*y*, −*z*+3/2; (xi) *x*−1/2, −*y*+1/2, *z*; (xii) −*x*+3/2, *y*, *z*−1/2; (xiii) −*x*+1/2, *y*−1/2, −*z*; (xiv) *x*, *y*−1/2, −*z*+1/2; (xv) *x*+1/2, −*y*, −*z*+3/2; (xvi) −*x*+1/2, *y*+1/2, −*z*+1.

## References

[bb1] Bruker (2002). *SMART*, *SAINT* and *SADABS* Bruker AXS Inc., Madison, Wisconsin, USA.

[bb2] Dolgikh, V. A. (1991). *Russ. J. Inorg. Chem. (Eng. Transl.)*, **36**, 1117–1129.

[bb3] Dowty, E. (2006). *ATOMS for Windows* Version 6.3. Shape Software, Kingsport, Tennessee, USA.

[bb4] Miletich, R. & Pertlik, F. (1998). *J. Alloys Compds*, **268**, 107–111.

[bb5] Sheldrick, G. M. (1997). *SHELXL97* University of Göttingen, Germany.

[bb6] Weil, M. (2005). *Acta Cryst.* C**61**, i103–i105. 10.1107/S0108270105028210 16210754

[bb7] Weil, M. (2007). *Z. Anorg. Allg. Chem.* **633**, 1217–1222.

[bb8] Weil, M. & Stöger, B. (2007). *Acta Cryst.* E**63**, i202.

